# Modifiable Cardiovascular Risk Factors and Hemorrhagic Presentation in Dural Arteriovenous Fistulas: A Systematic Review With Exploratory Quantitative Synthesis

**DOI:** 10.1002/brb3.71676

**Published:** 2026-10-09

**Authors:** Alejandro N. Santos, Sai Sanikommu, Bashar Dawoud, Josef Williams, Josiah J. Z. Sherman, Christoph Wipplinger, Jayro Toledo, Ahmed Abdelsalam, Victor M. Lu, Joshua Hanna, Robert M. Starke

**Affiliations:** ^1^ Department of Neurological Surgery University of Miami School of Medicine Miami Florida USA; ^2^ Department of Neurosurgery Cleveland Clinic Weston Florida USA

**Keywords:** alcohol, antithrombotic therapy, cardiovascular risk factors, dural arteriovenous fistula, hyperlipidemia, hypertension, intracranial hemorrhage, meta‐analysis, smoking, statins, systematic review

## Abstract

**Background:**

Dural arteriovenous fistulas (dAVFs) carry a significant risk of intracranial hemorrhage (ICH), yet most literature has focused on angiographic features as predictors. The influence of modifiable cardiovascular risk factors (CVRFs) such as hypertension, diabetes, smoking, alcohol use, dyslipidemia, obesity, and use of antithrombotic/anticoagulant agents on hemorrhagic presentation or follow‐up outcomes remains largely unexplored.

**Methods:**

We conducted a systematic review according to PRISMA 2020 guidelines. PubMed, Embase, and Scopus were searched through August 2025 for observational studies including at least 10 patients and reporting CVRFs in dAVFs in relation to hemorrhagic presentation or follow‐up hemorrhage. Crude effect estimates were extracted or reconstructed where possible. Quantitative pooling was considered exploratory and performed only when at least two studies reported reconstructable data for the same exposure and outcome.

**Results:**

Of 918 records screened, 31 full texts were reviewed, and 3 studies met inclusion criteria, totaling 543 patients. Two studies (*n* = 281) provided data for exploratory quantitative synthesis, whereas one hemorrhage‐only registry cohort (*n* = 262) was summarized narratively for post‐hemorrhage outcomes. Hypertension was present in 89 of 281 patients (31.7%) and hyperlipidemia in 61 of 281 (21.7%). Hypertension was not associated with hemorrhagic presentation (OR 0.89, 95% CI 0.47–1.68; *I* ^2^ = 0%). Hyperlipidemia estimates were highly heterogeneous (*I* ^2^ = 93.6%) and unsuitable for clinical inference. Findings regarding smoking, alcohol consumption, statins, and ACE inhibitors were sparse, unadjusted, and inconsistent.

**Conclusions:**

Evidence regarding modifiable cardiovascular risk factors in dAVFs is limited, retrospective, and vulnerable to confounding. Angiographic features remain the most reliable predictors of hemorrhagic presentation. Current systemic and therapeutic associations are exploratory and require validation in prospective multicenter studies.

## Graphical abstract

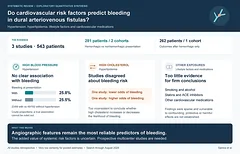

## 1 Introduction

Hemorrhagic risk in cerebrovascular disease is multifactorial (Hillen et al. 2003; Rinkel et al. 1998). In aneurysmal subarachnoid hemorrhage and primary intracerebral hemorrhage, associations with modifiable risk factors such as smoking, hypertension, and antithrombotic use are well established and strongly influence both risk and outcomes (An et al. 2017; Feigin et al. 2005; Hillen et al. 2003; Karhunen et al. 2021; Macdonald and Schweizer 2017). In cerebral cavernous malformations, some studies have suggested associations between systemic comorbidities and hemorrhage (Chen et al. 2021; Rauscher et al. 2023; Zuurbier et al. 2023; Zuurbier et al. 2022). Dural arteriovenous fistulas (dAVFs) represent approximately 10%–15% of intracranial vascular malformations and can present with a spectrum of symptoms ranging from benign tinnitus to aggressive neurological deficits and intracranial hemorrhage (ICH) (Abecassis et al. 2022; Gross and Du 2012; Sanchez et al. 2023). To date, hemorrhagic risk stratification in dAVFs has been almost exclusively driven by angiographic features, including Borden and/or Cognard grade, cortical venous drainage, and lesion location (Cognard et al. 1997; Cognard et al. 1995; Gandhi et al. 2012; Shah et al. 2012). In contrast, the influence of systemic comorbidities and modifiable cardiovascular risk factors (CVRFs), such as hypertension, diabetes, smoking, obesity, and dyslipidemia, remains poorly defined. Medication exposures, including antiplatelet, anticoagulant, and cardiovascular therapies, have been reported only sporadically and should be considered exploratory. Only a handful of small observational studies have reported these associations in patients with dAVFs, and no systematic review has comprehensively synthesized the available evidence (Gross et al. 2013; Koch et al. 2021; Li et al. 2015). To address this limitation, we performed a systematic review of published studies reporting modifiable cardiovascular risk factors in patients with dAVFs presenting with or without hemorrhage, with quantitative pooling considered an exploratory secondary analysis. Our findings highlight the scarcity of data and underscore the need for larger collaborative efforts. CONDOR (the CONsortium for Dural Arteriovenous Fistula Outcomes Research), an international multi‐institutional registry, provides a unique opportunity to investigate these questions in a large and diverse cohort (Abecassis et al. 2022; Chen et al. 2022; Samaniego et al. 2022). The aim of this study was to systematically characterize the available evidence regarding associations between modifiable cardiovascular risk factors and hemorrhagic presentation in dAVFs and to identify priorities for future studies.

## 2 Materials and Methods

This systematic review and meta‐analysis were conducted in accordance with the Preferred Reporting Items for Systematic Reviews and Meta‐Analyses (PRISMA) 2020 guidelines. The protocol was prospectively registered in the PROSPERO international prospective register of systematic reviews (registration number: CRD420251143231). As this study was based solely on analysis of previously published data, institutional review board approval and informed consent were not required.

### 2.1 Literature Search

A comprehensive search was performed in PubMed/MEDLINE, Embase, and Scopus from database inception through August 2025. The search strategy combined controlled vocabulary terms and free‐text keywords related to dAVF, hemorrhage, and modifiable cardiovascular risk factors, including hypertension, diabetes, smoking, alcohol consumption, obesity, hyperlipidemia, and the use of antiplatelet or anticoagulant therapies. Two reviewers (AS and BD) independently screened all titles and abstracts for eligibility, with full texts of potentially relevant articles subsequently assessed against prespecified inclusion and exclusion criteria. Disagreements were resolved by consensus or, when necessary, adjudicated by a third reviewer (RMS). Eligible studies were observational cohort or case–control designs, either retrospective or prospective, that reported on modifiable cardiovascular risk factors or systemic comorbidities in patients with dAVF, with outcomes of interest including hemorrhagic presentation at diagnosis and hemorrhage during follow‐up. Studies were excluded if they were case reports, conference abstracts, editorials, or reviews, if they included fewer than 10 patients, if they focused exclusively on angiographic predictors without reporting systemic comorbidities, or if they described only peri‐ or post‐procedural hemorrhagic complications rather than the natural history of dAVF. To ensure completeness, reference lists of all included articles and relevant reviews were manually screened. Only human studies published in or translated into English were considered. The PRISMA flowchart of the study selection process is presented in Figure 1 Figure 2.

**FIGURE 1:**
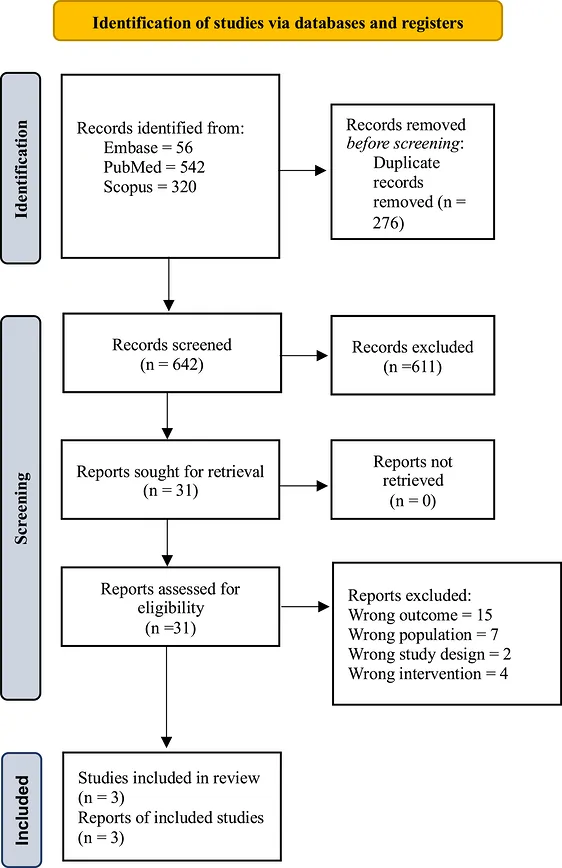
PRISMA flowchart of study selection.

**FIGURE 2:**
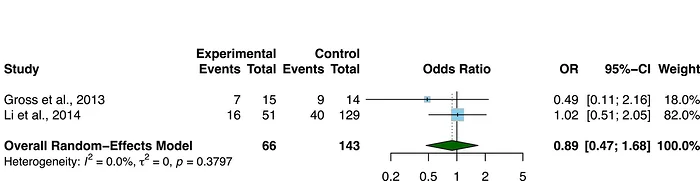
Forest plots showing pooled meta‐analysis of hypertension and odds of hemorrhage. Odds ratios (ORs) with 95% confidence intervals (CIs) are presented using random‐effects models; squares represent individual study estimates weighted by inverse variance, horizontal lines represent 95% CIs, and diamonds indicate the pooled summary effect. Heterogeneity across studies is expressed using *I* ^2^ statistics, *τ* ^2^, and *p*‐values.

### 2.2 Data Extraction

Two reviewers independently extracted data from selected articles using a standardized form. Extracted variables included study design, year, country, sample size, patient demographics, angiographic characteristics (Borden/Cognard grade, cortical venous drainage, fistula location), presence of cardiovascular risk factors (hypertension, diabetes, smoking, alcohol, obesity, dyslipidemia), use of antiplatelet or anticoagulant therapy, and clinical outcomes (hemorrhagic presentation, incident hemorrhage during follow‐up, mortality, and functional outcome). Reported effect measures (odds ratios, hazard ratios, risk ratios, or crude event counts) were recorded along with 95% confidence intervals and adjustment variables when available. When possible, we reconstructed 2 × 2 contingency tables categorizing patients as exposed with hemorrhage, exposed without hemorrhage, unexposed with hemorrhage, and unexposed without hemorrhage, to perform meta‐analyses directly from crude event counts. If studies lacked sufficient data for pooling, their findings were instead summarized narratively to contextualize the available evidence. Any disagreements were discussed and, if unresolved, adjudicated by a third reviewer (RMS). The risk of bias of included studies was qualitatively assessed, taking into account study design, sample size, and reporting of confounders, using the ROBINS‐I framework. Results are summarized in Figure S1.

### 2.3 Statistical Analysis

The association between individual cardiovascular risk factors and ICH was assessed using 2 × 2 contingency tables, considering both hemorrhagic presentation at diagnosis and incident hemorrhage during follow‐up when reported. For each study, patients were classified according to the presence or absence of a given risk factor and whether they presented with ICH or not. This created four categories: patients with the risk factor who presented with ICH, patients with the risk factor who did not present with ICH, patients without the risk factor who presented with ICH, and patients without the risk factor who did not present with ICH. From these cell counts, crude odds ratios (ORs) with 95% confidence intervals (CIs) were calculated, with the formula OR = (*a* × *d*) / (*b* × *c*). Quantitative pooling was considered an exploratory secondary analysis and was conducted when at least two studies reported reconstructable data for the same exposure and outcome. For the meta‐analysis, we computed pooled unadjusted odds ratios (ORs) with their 95% confidence intervals (CIs) for all outcomes using a random‐effects model with the Mantel–Haenszel method. When zero events or zero CIs were reported in a study, a continuity correction was applied to enable analysis of those studies. Heterogeneity across studies was assessed with the *Q* test and quantified with the *I* ^2^ statistic, with values ≥50% considered to indicate substantial heterogeneity. Given the limited number of eligible studies, only risk factors with at least two datasets, such as hypertension and hyperlipidemia were included in quantitative synthesis. For other risk factors such as diabetes, smoking, alcohol, anticoagulant use and antiplatelet therapy, the data could not be meta‐analyzed and findings were therefore summarized narratively. This approach preserved transparent reporting of crude event counts while treating pooled estimates as exploratory and emphasizing descriptive synthesis when quantitative analysis was not appropriate. Publication bias was not assessed because fewer than 10 studies were available for any exposure, making funnel‐plot interpretation and formal small‐study‐effect testing unreliable. All analyses and plots were generated using R statistical software (version 4.2.0) and R Studio. The risk of bias for included studies was assessed qualitatively, considering study design, sample size, and reporting of confounders; certainty of evidence for each exposure was further evaluated using the GRADE framework.

## 3 Results

### 3.1 Study Identification

Our systematic search retrieved 918 records across PubMed, Embase, and Scopus. After removal of 276 duplicates, 642 unique titles and abstracts were screened. Of these, 611 were excluded for not meeting eligibility criteria. Exclusion criteria regarded studies reporting only angiographic predictors without systemic comorbidities, case reports, reviews, conference abstracts, sample sizes of <10 patients, reports of peri‐ or post‐procedural hemorrhage rather than natural history or overlapping datasets. The latter left 31 full‐text articles for detailed review. Ultimately, three studies met inclusion criteria and provided usable data: Gross et al. (2013), Li et al. (2015), and Koch et al. (2021) (Figure 1). Gross and Li offered sufficient data to reconstruct 2×2 contingency tables for exposures including hypertension, hyperlipidemia, diabetes, smoking, alcohol use, and medications such as statins, ACE inhibitors, beta‐blockers, calcium channel blockers, aspirin, warfarin, and SSRIs. Meta‐analysis was feasible only for hypertension and hyperlipidemia, as these were reported in more than one study. Other exposures were summarized narratively. Koch et al. included only patients who had already presented with hemorrhage and was therefore incorporated narratively to describe post‐hemorrhage outcomes rather than predictors of hemorrhagic presentation. No additional eligible studies were identified through manual reference screening.

### 3.2 Study Characteristics

The three studies, published between 2013 and 2021, were retrospective cohort analyses conducted in North America (Gross, Koch) and Asia (Li). Together, they included 543 patients with dAVF, of whom 281 from Gross and Li were analyzable for associations between modifiable risk factors and hemorrhage. The additional 262 patients in Koch's CONDOR registry study all presented with hemorrhage and were therefore used to provide post‐hemorrhage outcome data rather than predictors of hemorrhagic presentation. Gross et al. (2013) analyzed 45 patients at a single US institution, systematically recording comorbidities and medication use. Li et al. (2015) reviewed 236 patients from Beijing Tiantan Hospital, with 56 (23.7%) presenting with hemorrhage. This study captured lifestyle factors such as smoking and alcohol consumption alongside angiographic data. Koch et al. (2021) reported 262 patients with hemorrhagic dAVF from the multicenter CONDOR registry, focusing on outcomes after bleeding. Across studies, mean patient age ranged from 48 to 59 years, with male predominance of 65%–70%. Comorbidities and exposures included hypertension, diabetes, dyslipidemia, smoking, alcohol, obesity (when reported), and medications. The overall risk of bias was moderate to serious, largely due to retrospective design, small or selected cohorts, heterogeneity in variable definitions, and limited adjustment for confounding. Koch et al. had serious risk of bias for the review question of hemorrhagic presentation because the cohort was restricted to patients who had already hemorrhaged.

From the two analyzable cohorts, hypertension was present in 89 of 281 patients (31.7%), with 23 (25.8%) presenting with hemorrhage compared with 49 of 192 (25.5%) without hypertension. Hyperlipidemia was recorded in 61 patients (21.7%), with 17 (27.9%) presenting with hemorrhage versus 55 of 220 (25.0%) without. Diabetes was available only from Li's cohort (27 patients, 11.4%), with 3 (11.1%) presenting with hemorrhage. Smoking was reported in 71 patients (30.1%), with 30 (42.3%) presenting with hemorrhage compared with 36 of 165 (21.8%) non‐smokers. Alcohol use was reported in 72 patients (30.5%), of whom 24 (33.3%) presented with hemorrhage versus 32 of 164 (19.5%) without. Medication exposures, most systematically reported by Gross, included statins (2/15 [13.3%] hemorrhage vs. 14/30 [46.7%] without), ACE inhibitors (0/9 [0%] vs. 16/36 [44.4%]), aspirin (1/9 [11.1%]), warfarin (3/5 [60%]), and SSRIs (2/5 [40%]). Numbers were too small for most therapies to draw firm conclusions (Table 1).

**TABLE 1:** Baseline characteristics of the studies included.

Study	Study design (period)	Country	Total patients	Age, year	Female, %	Modifiable factors studied	Specific factor	Exposed: hemorrhage, *n*	Exposed: no hemorrhage, *n*	Unexposed: hemorrhage, *n*	Unexposed: no hemorrhage, *n*
Gross et al. (2013)	Retrospective (2004–2012)	USA	45	59.6 ± 14.8	42	Hypertension; hyperlipidemia; beta‐blocker; ACE inhibitor; CCB; statin; aspirin; warfarin; SSRI	Hypertension (exposed *N* = 22; unexposed *N* = 23)	7	15	9	14
							Hyperlipidemia (exposed *N* = 15; unexposed *N* = 30)	2	13	14	16
							Beta‐blocker (exposed *N* = 11; unexposed *N* = 34)	3	8	13	21
							ACE inhibitor (exposed *N* = 9; unexposed *N* = 36)	0	9	16	20
							CCB (exposed *N* = 7; unexposed *N* = 38)	1	6	15	23
							Statin (exposed *N* = 15; unexposed *N* = 30)	2	13	14	16
							Aspirin (exposed *N* = 9; unexposed *N* = 36)	1	8	15	21
							Warfarin (exposed *N* = 5; unexposed *N* = 40)	3	2	13	27
							SSRI (exposed *N* = 5; unexposed *N* = 40)	2	3	14	26
Koch et al. (2021)	Retrospective (1990–2017)	USA	262	59 ± 13	30	Hypertension; anticoagulants; smoking	Not reconstructable	NR	NR	NR	NR
Li et al. (2014)	Retrospective (2009–2013)	China	236	48 ± 13	35.2	Hypertension; hyperlipidemia; diabetes; smoking; alcohol consumption	Hypertension (exposed *N* = 67; unexposed *N* = 169)	16	51	40	129
							Hyperlipidemia (exposed *N* = 46; unexposed *N* = 190)	15	31	41	149
							Diabetes (exposed *N* = 27; unexposed *N* = 209)	3	24	53	156
							Smoking (exposed *N* = 71; unexposed *N* = 165)	30	41	36	129
							Alcohol consumption (exposed *N* = 72; unexposed *N* = 164)	24	48	32	132

*Note*: The four outcome columns show raw patient counts. Exposure‐group denominators are reported once in the “Specific factor” column.

NR, not reported or not reconstructable from the published article; CCB, calcium channel blocker; SSRI, selective serotonin reuptake inhibitor.

### 3.3 Quantitative Synthesis and Meta‐Analysis

Meta‐analysis was feasible for hypertension and hyperlipidemia. For hypertension, pooled data from Gross and Li showed no significant association with hemorrhagic presentation (OR 0.89, 95% CI 0.47–1.68), with negligible heterogeneity (*I* ^2^ = 0%). For hyperlipidemia, the two studies produced markedly discordant estimates, with substantial heterogeneity (*I* ^2^ = 93.6%) and extreme imprecision. Therefore, no clinically interpretable pooled conclusion was drawn; individual study results are emphasized, and the exploratory pooled estimate is provided only in the Supplementary Material for transparency. Sensitivity analyses were not performed because only two studies were available. Publication bias was not assessed because fewer than 10 studies were available. Certainty of evidence for these estimates was graded as very low using the GRADE framework because of risk of bias, inconsistency, and imprecision (Table 2).

**TABLE 2:** Certainty of evidence (GRADE) for pooled meta‐analyses of modifiable cardiovascular risk factors in relation to hemorrhagic presentation in dAVFs.

Exposure	Starting certainty (observational)	Risk of bias	Inconsistency	Imprecision	Indirectness / Pub bias	Final certainty	Grade
Hypertension	Low	↓	↓	↓	(↓/–)	**Very low**	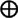 ◯◯◯
Hyperlipidemia	Low	↓	↓	↓	(↓/–)	**Very low**	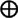 ◯◯◯

*Note*: Hypertension and hyperlipidemia were the only exposures with sufficient data for pooling; both were graded as very low certainty due to risk of bias, inconsistency, and imprecision.

GRADE: 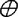
◯◯◯: very low; 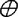 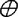
◯◯: low; 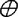 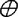 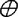
◯: moderate; 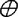 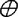 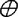 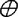: high.

### 3.4 Narrative Review

For other risk factors, data were insufficient for pooling. Gross et al. reported lower crude hemorrhage proportions among patients receiving statins and ACE inhibitors, but these findings were based on very small, unadjusted groups and do not establish protective effects (Gross et al. 2013). Antiplatelet and anticoagulant use showed no consistent associations, though warfarin users had numerically higher hemorrhage rates (Gross et al. 2013). Li et al. (2015) identified smoking and alcohol use as strongly associated with hemorrhagic presentation in univariate analyses, but these did not retain significance once angiographic predictors were accounted for. Venous drainage pattern remained the dominant factor (Li et al. 2015). Koch et al. (2021) analyzed only patients who had already presented with hemorrhage. Its finding that anticoagulant therapy independently predicted poor functional outcome therefore pertains to prognosis after hemorrhage and cannot be interpreted as evidence that anticoagulation increases the likelihood of hemorrhagic presentation.

## 4 Discussion

Hemorrhagic risk in cerebrovascular disease is multifactorial, shaped by both local vascular features and systemic comorbidities (Hillen et al. 2003; Rinkel et al. 1998). In aneurysmal subarachnoid hemorrhage, cavernous malformations, and primary intracerebral hemorrhage, modifiable cardiovascular risk factors (CVRFs) such as hypertension, diabetes, dyslipidemia, and smoking have been shown to influence both bleeding risk and outcomes (An et al. 2017; Chen et al. 2021; Feigin et al. 2005; Hillen et al. 2003; Karhunen et al. 2021; Macdonald and Schweizer 2017; Rauscher et al. 2023; Zuurbier et al. 2023; Zuurbier et al. 2022). These associations have guided risk stratification, prevention strategies, and therapeutic decision‐making in those settings. In contrast, dural arteriovenous fistulas (dAVFs), which account for 10%–15% of intracranial vascular malformations, remain comparatively underexplored (Abecassis et al. 2022; Gross and Du 2012; Sanchez et al. 2023). Current practice of risk assessment continues to rely almost exclusively on angiographic features, such as venous reflux patterns and lesion location (Cognard et al. 1997; Cognard et al. 1995; Gandhi et al. 2012; Shah et al. 2012).

The present review sought to address this gap by evaluating the role of systemic comorbidities and therapies. Across the limited available literature, only three studies provided extractable data, underscoring the scarcity of evidence. Collectively, these cohorts highlight the paucity of data regarding CVRFs. The largest series found that smoking and alcohol use were more common among hemorrhagic presentations in univariate analyses, but these associations did not persist after adjustment for angiographic factors (Li et al. 2015). A smaller cohort reported lower crude hemorrhage proportions with statins and ACE inhibitors, but the data were insufficient to establish protective effects (Gross et al. 2013). Registry data from a multicenter cohort added complementary evidence, suggesting that anticoagulant use and older age may worsen outcomes once hemorrhage has occurred (Koch et al. 2021). The exploratory pooled analysis found no significant association between hypertension and hemorrhagic presentation. Hyperlipidemia estimates were highly heterogeneous and too imprecise for clinical inference.

The narrative findings regarding smoking, alcohol, and cardiovascular therapies remain exploratory and vulnerable to uncontrolled confounding (Koch et al. 2021; Li et al. 2015). Importantly, risk factors associated with hemorrhagic presentation must be distinguished from factors associated with outcomes after hemorrhage. Because Koch et al. (2021) included only hemorrhagic cases, its anticoagulation finding applies to post‐hemorrhage prognosis and not to the occurrence of bleeding. These observations align with broader cerebrovascular evidence (Apostolaki‐Hansson et al. 2021; Rinkel et al. 1997). In cavernous malformations, antithrombotic therapy has been associated with reduced hemorrhagic risk (Gomez‐Paz et al. 2020; Zuurbier et al. 2023), while systemic conditions such as obesity may influence long‐term outcomes (Chen et al. 2021). In arteriovenous malformations, hypertension as well as lifestyle exposures such as smoking have similarly been implicated in rupture risk (Dalyai et al. 2014; Langer et al. 1998). By analogy, dAVFs may also be influenced by systemic comorbidities, potentially in interaction with angioarchitectural severity. No study to date, however, has been adequately powered to formally test such interactions. Given the rarity of dAVFs and fragmentation of available data, robust answers will require large‐scale, collaborative efforts. Multicenter registries such as CONDOR are uniquely positioned to incorporate standardized collection of comorbidities and therapies, enabling rigorous evaluation of their contribution to both hemorrhagic presentation and prognosis. Angiographic features remain the most reliable predictors of bleeding (Koch et al. 2021; Ma et al. 2025; van Dijk et al. 2002). Whether systemic risk factors or cardiovascular therapies provide additional prognostic information remains uncertain. Understanding these associations could ultimately expand prognostic models and inform preventive strategies in clinical practice.

### 4.1 Limitations

This analysis is constrained by the very small number of eligible reports, of which only two provided data suitable for pooling. The limited sample size increases the likelihood of type II error and limits generalizability. All included studies were retrospective, with inconsistent definitions of comorbidities, incomplete reporting, and variable adjustment for angiographic severity, introducing potential misclassification and residual confounding. Substantial heterogeneity was observed for hyperlipidemia, making the pooled estimate unsuitable for clinical inference and reinforcing the need to emphasize individual study results. Random‐effects modeling does not resolve clinical or methodological heterogeneity when only two studies are available. ROBINS‐I identified moderate‐to‐serious risk of bias across the included evidence. Selection bias is likely, as the included populations were drawn from tertiary centers and specialized registries. The Koch registry included only hemorrhagic cases and therefore informed prognosis after hemorrhage but not predictors of hemorrhagic presentation. Most importantly, the very small number of analyzable patients renders any apparent protective or harmful associations fragile. Subgroup analysis, sensitivity analysis, and assessment of publication bias were not feasible. These findings should therefore be interpreted cautiously, considered hypothesis‐generating, and viewed primarily as a foundation for larger, prospective multicenter studies to clarify the role of systemic risk factors in dAVF‐related hemorrhage.

## 5 Conclusion

This systematic review highlights the scarcity of data on modifiable cardiovascular risk factors in dural arteriovenous fistulas. Angiographic features remain the strongest predictors of hemorrhage, while observations regarding smoking, alcohol, dyslipidemia, and medications are sparse, unadjusted, and vulnerable to confounding. These findings are exploratory and do not establish protective or harmful effects. Prospective multicenter studies are needed to clarify whether these factors provide information beyond established angiographic predictors.

## Author Contributions

**Bashar Dawoud**: Writing – review and editing. **Sai Sanikommu**: methodology, formal analysis. **Robert M. Starke**: supervision, writing – review and editing, project administration. **Alejandro N. Santos**: conceptualization, validation, writing – review and editing, writing – original draft, investigation. **Josiah J. Z. Sherman**: writing – review and editing. **Christoph Wipplinger**: writing – review and editing. **Ahmed Abdelsalam**: writing – review and editing. **Jayro Toledo**: writing – review and editing. **Joshua Hanna**: writing – review and editing. **Victor M. Lu**: writing – review and editing. **Josef Williams**: writing – review and editing.

## Funding

RMS research is supported by the Codina Kadre Endowed Chair in Neurosurgery, the NREF, Joe Niekro Foundation, Brain Aneurysm Foundation, Bee Foundation, TAAF, Florida Department of Health James and Esther King Biomedical Research Program (21K02), and by the National Institutes of Health (R01NS111119‐01A1, UL1TR002736, KL2TR002737) through the Miami Clinical and Translational Science Institute. Its contents are solely the responsibility of the authors and do not necessarily represent the official views of the NIH.

## Ethics Statement

This study is a systematic review and meta‐analysis based exclusively on previously published data and did not involve direct interaction with human participants or access to identifiable patient information.

## Conflicts of Interest

The authors declare no conflicts of interest related to this work. RMS reports consulting and teaching agreements with Penumbra, Abbott, Balt, Kaneka, Medtronic, Stryker, Microvention, Integer PPF, InNeuro Co, VonVascular, Optimize Vascular, and Cerenovus.

## Supporting information




**Figure S1**: brb371676‐sup‐0001‐FigureS1.pdf

**Figure S2**: brb371676‐sup‐0002‐FigureS2.pdf
