# Supplementary material for: Modifiable Cardiovascular Risk Factors and Hemorrhagic Presentation in Dural Arteriovenous Fistulas: A Systematic Review With Exploratory Quantitative Synthesis
Source: Brain Behav. 2026 Oct 9;16(10):e71676. doi: 10.1002/brb3.71676 (PMC13653029; doi:10.1002/brb3.71676)
Supplement: Supplementary file 1 [file BRB3-16-e71676-s001.pdf]

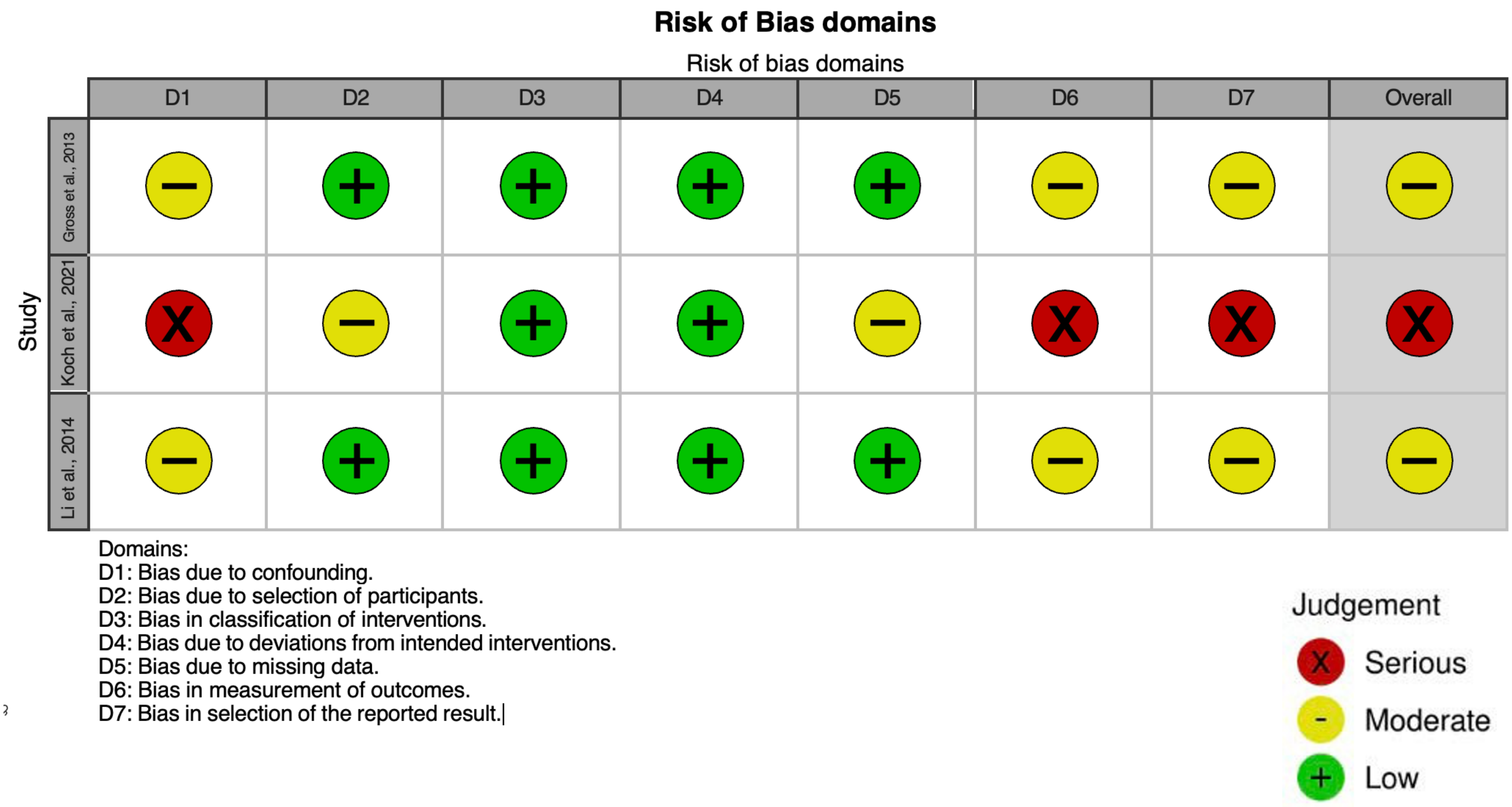

Supplementary Figure 1. Risk of Bias of Non-Randomized Studies. Risk of bias was assessed using the ROBINS-I tool across seven domains: confounding (D1), selection of participants (D2), classification of interventions (D3), deviations from intended interventions (D4), missing data (D5), measurement of outcomes (D6), and selection of the reported result (D7). Each domain was graded as low (green), moderate (yellow), or serious risk (red), with an overall judgment shown in the final column. Two studies (Gross et al., 2013; Li et al., 2014) were judged to have a moderate overall risk of bias, while one study (Koch et al., 2021) was rated as serious overall risk, primarily due to confounding, missing data, and selective reporting. Common concerns included moderate risk in measurement of outcomes and selection of reported results, highlighting methodological variability and the inherent limitations of observational evidence.
