# Supplementary material for: Modifiable Cardiovascular Risk Factors and Hemorrhagic Presentation in Dural Arteriovenous Fistulas: A Systematic Review With Exploratory Quantitative Synthesis
Source: Brain Behav. 2026 Oct 9;16(10):e71676. doi: 10.1002/brb3.71676 (PMC13653029; doi:10.1002/brb3.71676)
Supplement: Supplementary file 2 [file BRB3-16-e71676-s002.pdf]

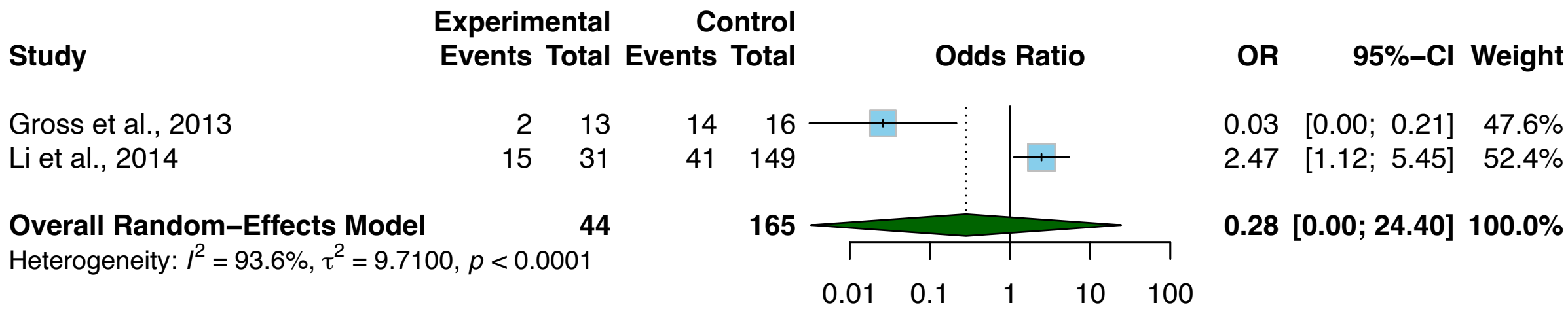

Supplementary Figure S2. Exploratory pooled analysis of hyperlipidemia and odds of hemorrhagic presentation in dural arteriovenous fistulas. Because only two markedly heterogeneous studies contributed data (I^2=93.6%) and the pooled confidence interval was extremely imprecise, this estimate is unsuitable for clinical inference and is presented only for transparency.
